# Live Attenuated *S*. Typhimurium Vaccine with Improved Safety in Immuno-Compromised Mice

**DOI:** 10.1371/journal.pone.0045433

**Published:** 2012-09-24

**Authors:** Balamurugan Periaswamy, Lisa Maier, Vikalp Vishwakarma, Emma Slack, Marcus Kremer, Helene L. Andrews-Polymenis, Michael McClelland, Andrew J. Grant, Mrutyunjay Suar, Wolf-Dietrich Hardt

**Affiliations:** 1 Institute of Microbiology, D-BIOL, ETH Zürich, Zürich, Switzerland; 2 School of Biotechnology, KIIT University, Bhubaneswar, Odisha, India; 3 Städtisches Klinikum München, München, Germany; 4 The Vaccine Research Institute of San Diego, San Diego, California, United States of America; 5 Department of Veterinary Medicine and Cambridge Infectious Diseases Consortium, University of Cambridge, Cambridge, United Kingdom; Indian Institute of Science, India

## Abstract

Live attenuated vaccines are of great value for preventing infectious diseases. They represent a delicate compromise between sufficient colonization-mediated adaptive immunity and minimizing the risk for infection by the vaccine strain itself. Immune defects can predispose to vaccine strain infections. It has remained unclear whether vaccine safety could be improved via mutations attenuating a vaccine in immune-deficient individuals without compromising the vaccine's performance in the normal host. We have addressed this hypothesis using a mouse model for *Salmonella* diarrhea and a live attenuated *Salmonella* Typhimurium strain (*ssaV*). Vaccination with this strain elicited protective immunity in wild type mice, but a fatal systemic infection in immune-deficient *cybb* ^−/−^ *nos2* ^−/−^ animals lacking NADPH oxidase and inducible NO synthase. In *cybb* ^−/−^ *nos2* ^−/−^ mice, we analyzed the attenuation of 35 *ssaV* strains carrying one additional mutation each. One strain, Z234 (*ssaV SL1344_3093*), was >1000-fold attenuated in *cybb* ^−/−^ *nos2* ^−/−^ mice and ≈100 fold attenuated in *tnfr1* ^−/−^ animals. However, in wt mice, Z234 was as efficient as *ssaV* with respect to host colonization and the elicitation of a protective, O-antigen specific mucosal secretory IgA (sIgA) response. These data suggest that it is possible to engineer live attenuated vaccines which are specifically attenuated in immuno-compromised hosts. This might help to improve vaccine safety.

## Introduction

Bacterial infections are a worldwide health burden. The rise of resistance against the current antibiotics and the limited availability of novel drug targets [1] have fuelled the interest in prophylactic approaches including live attenuated bacterial vaccines (LAV). In general, LAV are attenuated versions of the pathogen itself or closely related bacterial species and represent a delicate “compromise”. On the one hand, they must grow at inductive sites for providing antigens. On the other hand, the LAV must be sufficiently attenuated to avoid overt disease symptoms, in immune-proficient and (as far as possible) in immune-deficient individuals [2], [3]. The basic techniques for generating live attenuated vaccines are well established and should allow LAV design for many pathogenic bacteria. Nevertheless, until today there are only surprisingly few LAV approved for human use; Ty21a against *Salmonella* Typhi, CVD 103-HgR for *Vibrio cholerae*, and the BCG vaccine against *Mycobacterium tuberculosis* infections [4]–[7]. This scarcity of approved LAV is attributable at least in part to the safety concerns arising from the risk of fulminant infections which may be caused by the vaccine strains in immuno-compromised individuals, like those suffering from immunosuppressive infections (e.g. HIV) or genetic disease (e.g. CGD, chronic granulomatous disease). This safety problem could be solved, if one could identify mutations which specifically “super”-attenuate the LAV in immuno-compromised individuals without compromising its performance in the immune-proficient host. So far, it has remained unclear whether it is possible to design such a super-attenuated live attenuated vaccine (saLAV).

We have focused on vaccine design against non-typhoidal *Salmonella enterica* (NTS), i.e. *Salmonella enterica* subspecies 1 serovar Typhimurium (*S*. Typhimurium). NTS are a frequent cause of diarrheal disease and of severe systemic infections in the young and in immuno-compromised humans suffering from HIV infection, autoimmune diseases, malnutrition or a history of antimicrobial drugs or cancer chemotherapy. If untreated, such systemic NTS infection cause significant mortality and morbidity [8]–[10]. Antibiotics are routinely prescribed in these severe cases [11]. However, the lack of well controlled clinical studies of their therapeutic benefit and the spread of antibiotic resistant strains has raised interest in strategies for disease prevention, i.e. hygiene policies, reduction of zoonotic exposure and the design of vaccines [12].

Several experimental NTS vaccines have been developed using *S*. Typhimurium mutants and a mouse model for typhoid fever [13], [14]. Only a few, including WT05 (*aroC ssaV*), LH1160 (*phoP phoQ purB*), VNP20009 (*purI xyl msbB*), were tested in clinical trials. Strain WT05 was well tolerated in human volunteers except one subject who developed fever and malaise. There was no bacteremia observed in any of the healthy human volunteers, however the organism was shed in feces at high doses (10^9^) until up to 23 days p.i. LH1160 was equally tolerated by healthy human volunteers with no bacteremia or diarrhea except 2 out of 6 subjects developed transient fever and other discomfort which restored without antibiotic intervention. VNP20009 was tolerated at lower doses (10^6^), i.e., bacteria was cleared from the blood within 12 hours after intravenous administration, however, extended bacteremia was observed in few patients at dose range of ∼10^9^ cfu and they required antibiotic treatment. [15]–[17]. Of particular concern, all of the above mentioned strains showed incidents of vaccine strain-inflicted disease and in some cases antibiotic intervention was required to limit disease progression [15], [17]–[21]. One may speculate that these cases were attributable to underlying genetic predispositions rendering some individuals susceptible to overt infection. This would be in line with BCG vaccination studies which identified clinical complications in individuals with underlying genetic defects [22]. Similarly, studies of live attenuated *Shigella* vaccines yielded fulminant shigellosis in some vaccinees [23]–[26] and use of the typhoid fever vaccine Ty21a is contra-indicated in case of a known immune-deficiency [27]. Thus, balancing immunogenicity versus safety has remained challenging and none of the current experimental *S*. Typhimurium vaccines has been approved for general use. Novel approaches for improving LAV safety would be of significant interest.

Recently, we have established an experimental model for analyzing vaccination and protection against NTS diarrhea [28]–[30]. The model involves pretreatment of mice with 20 mg of streptomycin by oral gavage, one day prior to oral vaccination with ∼10^7^ cfu of an attenuated *S*. Typhimurium strain. This guarantees efficient gut colonization by the attenuated *Salmonella* strain and elicitation of a protective immune response. At day 40 after the vaccination, the mice were treated with 20 mg of ampicillin in order to eliminate the regrown microbiota and any remaining bacteria of the vaccine strain and challenged with ∼200 wt SB300, one day later [28], [29], [31]. We have previously reported that such per oral vaccination of C57BL/6 mice with the LAV *S*. Typhimurium *sseD*, a Type Three Secretion System - 2 mutant (TTSS-2), elicit an O-antigen specific sIgA and IgG response protecting from *S*. Typhimurium diarrhea in challenge infections [28]–[30]. TTSS-2 mutants like *ssaV* are highly attenuated [32] and guarantee the survival of the vaccinated “wild type” C57BL/6 mice. However, pilot experiments in immuno-compromised mice indicated that the *ssaV* strain was not safe enough, causing lethal infections in *cybb* ^−/−^ *nos2* ^−/−^ mice (see below). These mice are lacking two key elements of the antibacterial defense, the inducible NO synthase and the NADPH oxidase, a reactive oxygen species generating enzyme [33]–[35]. In human CGD patients, NADPHox deficiency is known to increase susceptibility towards many bacteria, including NTS [10], [36]. Using the *S*. Typhimurium diarrhea model, we asked whether it might be possible to identify additional mutations which turn *ssaV* into a saLAV. Such a strain should ideally display attenuated virulence in immuno-compromised mice but should retain vaccination potency in wt animals. A screen of 35 site-directed double mutants identified one strain (Z234, *ssaV SL1344_3093*) which fulfilled these criteria. This proves the concept that saLAV can be generated and may have implications for improving vaccine safety with respect to immuno-compromised hosts.

## Results

### Identification of a live attenuated *S.* Typhimurium vaccine strain with improved safety features in *cybb* ^−/−^ *nos2* ^−/−^ mice

In a murine model for *S*. Typhimurium diarrhea, a *ssaV* mutant lacking a structural protein of the Type III Secretion System-2 (TTSS-2), was a safe vaccine eliciting protective mucosal immunity in wt C57BL/6 mice [28]–[30], but causes lethal infection in isogenic *cybb* ^−/−^ *nos2* ^−/−^ animals (see below). We hypothesized that one could circumvent this problem if it is possible to identify an additional mutation attenuating the vaccine strain in *cybb* ^−/−^ *nos2* ^−/−^ mice.

To test this hypothesis, we generated a set of 35 site-directed mutants of *ssaV* (Table S1). The second gene to be mutated was selected from a collection of known or putative virulence associated genes. Furthermore, every double mutant carried one out of seven different “barcode sequences” (WITS, wild type isogenic tagged strains [37]) which enabled the real-time PCR-based quantification of a given strain in mixtures of up to seven differentially tagged strains (Materials and Methods). For screening of the mutants, we established a co-infection protocol (Figure S1) allowing the analysis of 6 mutants along with the isogenic background strain *ssaV* (M2735; *ssaV::cat*) per mouse.

Attenuation of the mutant strains was analyzed in *cybb* ^−/−^ *nos2* ^−/−^ mice (C57BL/6 background; 3 animals per group) infected with a 1∶1∶1∶1∶1∶1∶1 mixture of the seven strains (*ssaV* +6 mutants; 5×10^7^ cfu in total by gavage). We monitored the intestinal colonization efficiency via fecal pellets retrieved at day 1 and the organ loads in the mesenteric lymph nodes (MLN) and the spleen at day 4 p.i. (Materials and Methods). Real-time PCR based quantification of the tags revealed the level of attenuation of the individual mutants. In particular, we were interested in identifying mutants strongly attenuated in the MLN and the spleen of the *cybb* ^−/−^ *nos2* ^−/−^ mice, while retaining wt levels of intestinal colonization.

**Figure 1:**
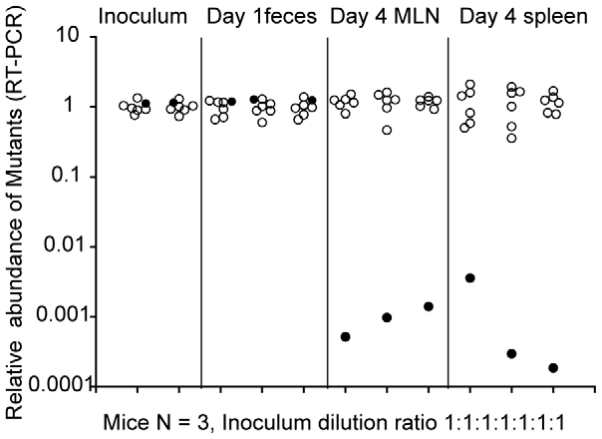
Screening experiment identifying Z234. Streptomycin pre-treated *cybb* ^−/−^ *nos2* ^−/−^ mice (n = 3) were co-infected with a 1∶1∶1∶1∶1∶1∶1 mixture of 6 different double mutants including Z234 (WITS tag 21) and the isogenic parental strain M2735 (*ssaV::cat;* WITS 17) and; *ssaV STM2616* (WITS 1), *ssaV STM2998* (WITS 2), *ssaV STM2011* (WITS11), *ssaV STM2231* (WITS13), *ssaV STM2570* (WITS19). Real-time PCR analysis of the sequence tags established the relative abundance of Z234 (black symbols) as compared to the parental strain and the five other double-mutants analyzed in these mice (open symbols).

35 strains were analyzed (Table S1). Interestingly, one strain (Z234; *ssaV SL1344_3093*) lacking a putative monoamine oxidase was 1000-fold attenuated in MLN and spleen colonization while growth in the gut lumen did not differ significantly from *ssaV* (Figure 1). This phenotype was intriguing because Z234 may have at least two features of a saLAV: Efficient growth in the gut lumen should facilitate efficient antigen sampling from the gut lumen (e.g. to lamina propria and lymph nodes) and the strong attenuation at systemic sites should improve “safety” in iNOS and NADPHoxidase deficient hosts, at least in the mouse model.

### Z234 is specifically super-attenuated in *cybb* ^−/−^ *nos2* ^−/−^, but not in wt C57BL/6 mice

The attenuation of Z234 in *cybb* ^−/−^ *nos2* ^−/−^ mice was verified in a standard 1∶1 competition experiment. In line with our initial observations, equivalent loads of *ssaV* and Z234 (C.I. = 1) were detected in the feces of *cybb* ^−/−^ *nos2* ^−/−^ mice at day 1 p.i. while Z234 was more than 1000-fold attenuated in MLN and spleen colonization (p<0.05; CI<0.001; Figure S2A) at day 4 post infection.

Equivalent 1∶1 competition experiments were performed to assess the performance of Z234 in wt C57BL/6 mice (Figure S2B). Again, *ssaV* and Z234 showed comparable gut colonization levels (C.I. = 1.43; p, not significant (N.s.)). It should be noted that neither Z234 nor M2735 colonized the spleen of wt C57BL/6 mice. This is expected, because TTSS-2 deficiency is well known to strongly attenuate systemic spread of *Salmonella* spp. in wt animals [29], [32], [38]. However, Z234 colonized the MLN of wt mice as efficiently as *ssaV* (Figure S2B; C.I. = 1.03; p, N.s.). This was a striking difference to the phenotype of Z234 in *cybb* ^−/−^ *nos2* ^−/−^ animals (compare MLN data from Figure S2A and S2B).

**Figure 2:**
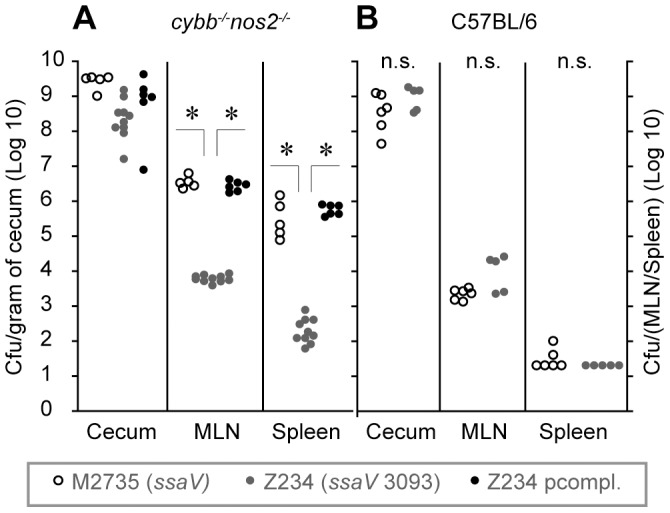
Verification of the attenuation of Z234. Streptomycin treated *cybb* ^−/−^ *nos2* ^−/−^ (2A) or C57BL/6 (2B) mice were infected either with *ssaV* (open), Z234 (grey) or Z234 p_compl._ (black; 5×10^7^ cfu by gavage; n = 5–10 mice). Bacterial loads in the cecum contents, the MLN and the spleens were quantified by plating at day 4 post infection; *, statistically significant (p <.05; Mann-Whitney U-test).

To further validate the attenuated phenotype, *cybb* ^−/−^ *nos2* ^−/−^ mice were infected with just one strain at a time, i.e. *ssaV*, Z234 or a complemented version thereof (Z234 p_compl._; Materials and Methods). In *cybb* ^−/−^ *nos2* ^−/−^ mice, Z234 could efficiently colonize the cecal lumen, but was >1000-fold attenuated in MLN and spleen colonization (Figure 2A). Furthermore, plasmid based complementation (Z234 p_compl._) resulted in phenotypic reversion and Z234 p_compl._ colonized the gut, the MLN and the spleen of *cybb* ^−/−^ *nos2* ^−/−^ mice as efficiently as the isogenic parent strain *ssaV*. Taken together, disruption of *SL1344_3093* in the background strain *ssaV* resulted in dramatic reduction of MLN colonization and of systemic *Salmonella* loads in severely immuno-compromised mice, suggesting that Z234 might be a safer live attenuated vaccine than *ssaV* if this type of host is encountered.

Equivalent infection experiments were employed to verify the performance of Z234 in wt C57BL/6 mice. Again, Z234 and M2735 were both incapable of colonizing the spleens of wt C57BL/6 mice (Figure 2B). However, both Z234 and M2735 colonized the cecal lumen and the MLN with high efficiency (Figure 2B; p, N.s.). Such comparable colonization of the gut associated mucosa and the draining lymph nodes suggested that Z234 might retain its immunogenic potential in wt mice, an important hallmark of any LAV.

### Z234 protects wt C57BL/6 mice in challenge infections with wt *S*. Typhimurium

Finally, we have analyzed the immunogenic potential of Z234 in wt mice. For this purpose, we employed the immunization-challenge protocol which was established recently using *S*. Typhimurium strains deficient of TTSS-2 as experimental LAV [29]. Using this protocol, the LAV was known to elicit within 40 days a serovar specific and robust mucosal secretory IgA response protecting efficiently from enteropathy in challenge infections with wt *S*. Typhimurium [28], [29].

Groups of wt C57BL/6 mice were immunized with *ssaV* (standard LAV; n = 9), Z234 (saLAV; n = 13) or PBS (neg. control; n = 4) and we analyzed fecal shedding during this vaccination period as a measure for antigen levels (Figure S3A). Both, *ssaV* and Z234 reached bacterial densities in the gut lumen of up to ∼10^9^ cfu/g as early as day 1 post immunization and bacterial loads declined at equivalent rates after day 16. Based on earlier work, this was attributable to the onset of the mucosal immune response and the subsequent out-competition of the LAV by the re-growing microbiota [29]. Stable, efficient and comparable bacterial colonization levels in the gut lumen should permit efficient antigen sampling by the mucosal immune system with respect to both strains.

At day 40 p.i., we sacrificed some of the immunized animals (*ssaV*, n = 4; Z234, n = 6) in order to analyze colonization levels and cecum histopathology. With both strains, cecum colonization had dropped to approx., 10^7^ cfu/g cecal contents, the MLN loads were lower than in the acute infection experiments (compare Figure S3B and Figure 2B) and the cecal mucosa did not show signs of disease (Figure S3B).

**Figure 3:**
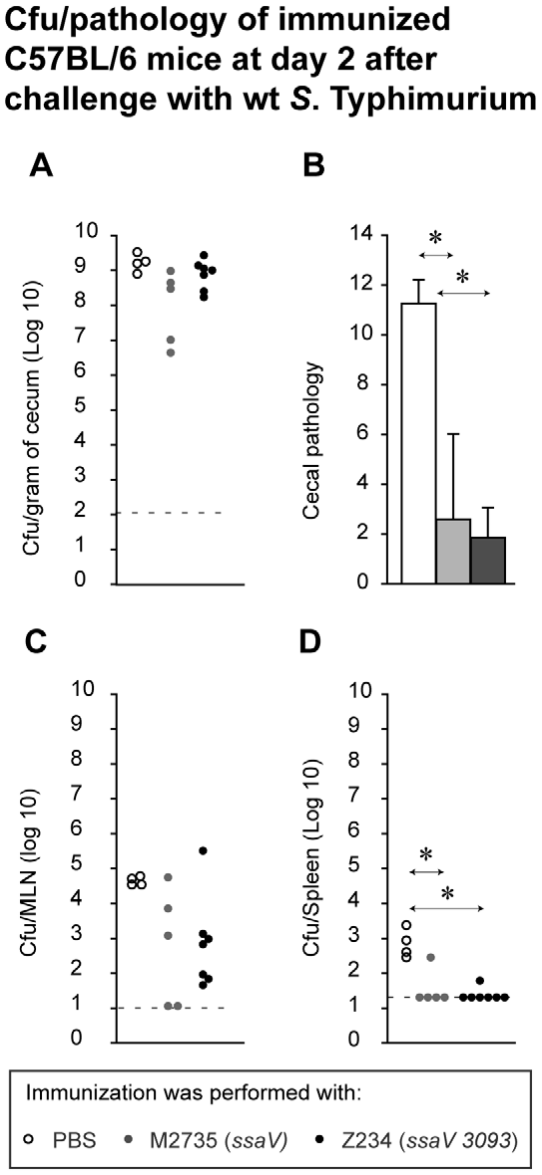
Vaccination-challenge analysis of the immunogenic potential of Z234. C57BL/6 mice were vaccinated with PBS (n = 4; open symbols), *ssaV* (5×10^7^ cfu; n = 9; grey) or Z234 (5×10^7^ cfu; n = 13; black). 40 days post immunization (PBS (n = 4); M2735 (n = 5); Z234 (n = 7)), mice were ampicillin-treated (20 mg by gavage) and challenged with wt SB300 (amp^r^, sm^r^) for two days.. Colonization of cecum (A), MLN (C), Spleen (D) and cecal pathology (B) after wt *S*. Typhimurium, day 2 post challenge was shown accordingly. Disease parameters (vaccination control data) were determined as in Figure S3B. Striped line: detection limit.

The remaining mice were analyzed for protection against a challenge with wt *S*. Typhimurium. At day 40, the immunized animals (*ssaV*, n = 5; Z234, n = 7; PBS, n = 4) were treated with 20 mg of ampicillin (i.g.; severely limits re-grown gut flora and any remaining LAV) and challenged with wt *S*. Typhimurium at day 41 (200 cfu by gavage). In line with earlier work, wt *S*. Typhimurium was able to colonize the lumen efficiently and reached the carrying capacity by day 2 post challenge in all three groups. As expected, the PBS controls suffered from severe enteropathy (median pathological score = 11.5; Figure 3B) which was marked by pronounced sub mucosal edema, PMN infiltration and loss of mucus filled vacuoles from the goblet cells and compromised epithelial integrity (Figure S4A). In contrast, 8/9 mice of the *ssaV*-immunized group and none of the Z234-immunized group displayed any signs of mucosal inflammation (p<0.05; median pathological score <3; Figure 3B and Figure S4B). Furthermore, spleen colonization by wt *S*. Typhimurium was significantly reduced in both vaccinated groups (p<0.05; Figure 3D). Thus, Z234 conferred equivalent levels of protection from *Salmonella*-inflicted disease as *ssaV*.

### O-antigen specific luminal and serum antibody responses in mice immunized with *ssaV* or Z234

**Figure 4:**
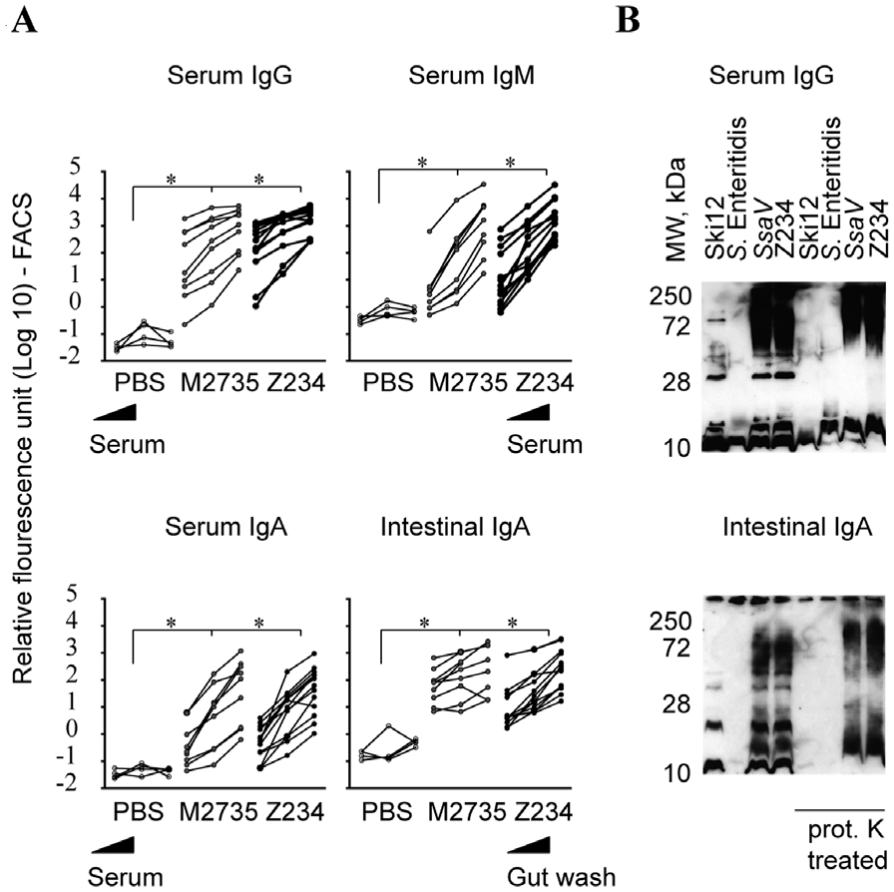
Antibody response (serum and intestinal wash) and specificity (western blot), post vaccination. (A) Antibody titers (serum IgG, IgM, IgA and intestinal IgA) as analyzed by bacterial FACS (Materials and Methods). Serial dilutions of serum/intestinal wash samples were analyzed. (B) Representative Western blot analysis of the antibody responses. Western blots of overnight cultures of Z234, M2735, SKI12 (O-antigen negative strain; Δ*wbaP*, SL1344) or *S*. Enteritidis wt (M1525; with or w/o proteinase K (PK) treatment) were developed using sera or gut luminal sIgA from mice immunized with Z234 or *ssaV* and by α-mouse IgG (for serum) or α-mouse IgA (for gut wash). N.s.: not significant; *, statistically significant (p<.05; Mann-Whitney U-test). —, Average detection limit.

Our earlier work had demonstrated that immune-protection was based on a robust O-antigen specific luminal sIgA which went along with serum IgA, IgM and IgG responses [29]. To further validate the immunogenic potency of Z234, we have therefore analyzed the antibody titers from the animals shown in Figure 3 and Figure S3B. Serum and gut wash samples were collected at the end of the 40 day vaccination procedure and antibody responses were quantified via a surface-coating FACS protocol and western blot. This assay relies on the specific antibody binding to O-antigens on the surface of a test bacterium, Z234 (Materials and Methods; [29]). The intestinal wash and serum samples from mice immunized with either *ssaV* or with Z234 exhibited equivalent titers of specific serum IgG, IgA and IgM and luminal sIgA (Figure 4A).

These data were confirmed by Western blot analyses. Bacteria from overnight cultures of *S*. Typhimurium strains (*ssaV*, Z234), wt *S*. Enteritidis (neg. control; has a different O-antigen) and the O-antigen deficient *S*. Typhimurium *wbaP* mutant (SKI12; neg. control) were treated with or without proteinase K, run on SDS-PAGE and immunoblotted with the respective mouse antibody sample (serum or intestinal wash) and subsequently tagged with IgG-, IgA- or IgM-specific secondary conjugates. In line with the data presented above, the *S*. Typhimurium O-antigen was the main macromolecule recognized by the antibodies from *ssaV*- and Z234-immunized mice (Figure 4B). Thus, Z234 and *ssaV* elicit comparable O-antigen-specific antibody responses.

### Safety assessment of Z234 in different immuno-deficient mouse lines

So far, we had focused on *cybb* ^−/−^ *nos2* ^−/−^ mice to assess the safety of Z234. However, it was unclear whether Z234 would also be super-attenuated in other immune-deficient mouse lines. This question would be of interest for any saLAV. Clinical reports on *S*. Typhimurium based vaccines and their impact on immuno-compromised people are limited. However, the *S*. Typhi vaccine Ty21a is contraindicated for people with severe antibody deficiencies, partial or complete T cell defects, impaired phagocytic function, etc [27]. Moreover, non-typhoidal *Salmonella* spp. (*S*. Typhimurium and *S*. Enteritidis) have been notorious for systemic infections in immuno-compromised children and adults [9], [10], [39], [40] (see also Reference S1). Similarly, in murine typhoid fever models, the *S*. Typhimurium vaccine strains (*aroA*, *htra*, *htra aroD*) caused severe disease in various immuno-deficient animal models [41], [42].

Therefore, we investigated the capacity of Z234 to cause systemic disease in mouse models deficient for interferon gamma signaling (*ifng* ^−/−^, defective phagocyte activation and adaptive immune responses [43]–[45]), complement activity (C3^−/−^, opsonization, bacterial killing, adaptive responses [46], [47]), Tumor necrosis factor signaling (*tnfr* ^−/−^, innate and adaptive immunity [48], [49]), caspase-1 signaling (*casp-1* ^−/−^, innate immunity [50]–[52]) and recombination activation gene 1 (*rag1* ^−/−^, lack mature B- and T-cells [38]). This choice of knockout mice was based on the published hyper-susceptibility towards systemic infection by wt *S*. Typhimurium [53], [54].

**Figure 5:**
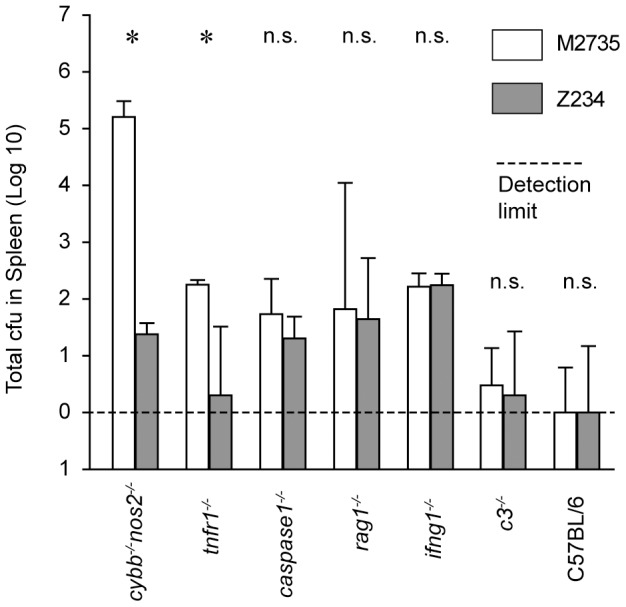
Safety of Z234 in immune-deficient mice. Animals were infected with a 1∶1 mixture (5×10^7^ cfu by gavage) of M2735 (open bars) and Z234 (gray bars) for four days. We analyzed loads in the spleens of *cybb* ^−/−^ *nos2* ^−/−^(B6), *tnfr1* ^−/−^ (B6), caspase1^−/−^ (B6), *rag1* ^−/−^ (B6), *ifng* ^−/−^ (BALB/c) and *c3* ^−/−^ (BALB/c). Data for wt C57BL/6 mice is shown for comparison. Bars: median +/− sdv. _*_, statistically significant (p <.05; Mann-Whitney U-test).

To assess safety, we performed standard competitive infection experiments with 1∶1 mixtures of *ssaV* and Z234. Generally, both strains colonized the gut efficiently at day 1 post infection (∼10^8^ cfu/g feces) in all mice and MLN loads ranged between ∼10^3^–10^4^ cfu by day 4, when the mice were sacrificed. The experiments revealed three different classes of phenotype with respect to the *SL1344_3093* mutation: a) specific attenuation; the systemic spread of Z234 was significantly reduced in *cybb* ^−/−^ *nos2* ^−/−^ mice (p<0.05; CI<0.001; Figure 5) and in *tnfr1* ^−/−^ mice (p<0.05; CI<0.01) suggesting that Z234 was specifically super-attenuated in these models. b) no specific attenuation: *rag1* ^−/−^ and *ifng1* ^−/−^ mice showed equally high susceptibility to both, Z234 and *ssaV*. c) sufficient attenuation by *ssaV* alone: In the C3^−/−^ mice and the wt C57BL/6 controls we did not detect significant systemic spread of either strain, suggesting that the *ssaV* mutation alone was attenuating *S*. Typhimurium sufficiently well. In conclusion, these data established that Z234 features increased safety in some (i.e. *cybb* ^−/−^ *nos2* ^−/−^, *tnfr1* ^−/−^), but not all types of immuno-deficiency, while retaining the full immunogenic potential in wild type hosts.

## Discussion

Here, we have performed a proof of principle study on the experimental *S*. Typhimurium diarrhea vaccine strain *ssaV* to establish whether safety can be enhanced in immuno-compromised hosts, i.e. *cybb* ^−/−^ *nos2* ^−/−^ mice. One out of 35 double mutants (i.e. Z234) screened in *cybb* ^−/−^ *nos2* ^−/−^ mice was specifically attenuated in these hosts but retained the immunogenic potential of the parental LAV (*ssaV*) in wt C57BL/6 mice. The wt mice responded with *S*. Typhimuirum specific antibodies (serum IgA, IgM, IgG and luminal sIgA) against the O-antigen. In our previous work with the parental LAV, we also demonstrated that protection against wt secondary infection was dependent on O-antigen-specific sIgA and required both T cells (using TCR β^−/−^/δ^−/−^) and B cells (JH^−/−^, IgA^−/−^) [29]. Furthermore, it had been shown that genetically susceptible C57BL/6 mice cannot be fully protected against a virulent challenge with passive transfer of *S*. Typhimurium specific antibodies alone [53], [55], indeed, (in contrast to naturally resistant mice) protection also required T cell activation [56]–[58]. Overall, Z234 elicited comparable host immune response (including the T-and B-cell requirement for full protection) as the parental LAV. However, in addition Z234 displayed key features of a saLAV, including specific super-attenuation in immuno-compromised hosts.

For the original screen, we had chosen *cybb* ^−/−^ *nos2* ^−/−^ mice, because this line was highly susceptible to fatal systemic spread of the parental LAV (*ssaV*) in our pilot experiments and moreover it displays a genetic defect commonly found in human CGD patients who are particularly prone to systemic NTS infection [10], [36]. Based on this observation, it was interesting to note that Z234 was specifically super-attenuated not only in the *cybb* ^−/−^ *nos2* ^−/−^ mice, but also in animals deficient for TNF signaling. On the other hand, mice with other types of immune-deficiency (*rag1* ^−/−^ and *ifng1* ^−/−^) were equally susceptible to *ssaV* and Z234. Thus, the safety of the saLAV was enhanced in some immuno-deficient hosts, and remained as safe as *ssaV* in others. The reasons for this will be an important topic for future research aimed at further improving the safety of this experimental vaccine.

In spite of its high level of specific attenuation, it was interesting to note that Z234 retained some residual capacity to colonize the MLN of *cybb* ^−/−^ *nos2* ^−/−^ mice. The residual population size in the MLN was quite similar to that observed for the parental LAV *ssaV* in wt C57BL/6 mice. Based on this correlative evidence, one may speculate that the *cybb/nos2* deficiency may open a new replicative niche in the host's lymph node cell populations which can be exploited by *ssaV*, but not by Z234. In line with this hypothesis, earlier work has attributed the failure of TNFR^−/−^ macrophages to control the growth of a TTSS-2 mutant to defects in NADPH oxidase trafficking and *cybb* mediated killing [49]. Phagocytic leukocyte populations, i.e., macrophages and PMN are key mediators of *nos2*- or *cybb*-mediated killing of bacteria via reactive nitrogen and/or oxygen species [33], [59]. Thus, we hypothesize that the disruption of *SL1344_3093* may disable the growth of Z234 in some *cybb* ^−/−^ *nos2* ^−/−^ phagocytic leukocyte population. Clearly, more work will be required to determine whether this mechanism may explain the specific super-attenuation of Z234 in *cybb* ^−/−^ *nos2* ^−/−^ mice.

Database searches and experimental infection data may provide clues about the molecular basis of *SL1344_3093* mediated specific super-attenuation. The protein encoded by *SL1344_3093* (identical to *STM3119* from LT2) is predicted to adopt a “hot dog fold” (cI00509; NCBI conserved domain database) and shares significant similarity with enoyl-CoA dehydratases. Homologs are found not only in *Salmonella* spp., but also in other pathogenic bacteria, like *Yersinia* spp., *Mycobacterium* spp., *Burkholderia* spp., *Legionella longbeachae*, *Mycoplasma* spp., *Coxiella burnetii* and *Corynebacterium pseudotuberculosis*. In several species, the conservation extends to the neighboring genes or the entire operon. For example, *Mycobacterium tuberculosis* shares the neighboring gene *citE* (a citrate lyase β subunit) and in *S*. Enteritidis, *Y. pestis* and *Y. pseudotuberculosis* the homology extends to a LysR-like transcriptional regulator (*SL1344_3095*, *S*.En *stmR*, *Yersinia* y2382) and an operon encoding a citrate lyase β subunit (*SL1344_3094*, *S*.En *citE*, *Yersinia y2383*/*ripC*), the enoyl-CoA dehydratase/hydratase (*SL1344_3093*, *S*.En *fkbR2*, *Yersinia y2384*/*ripB*) and a butyryl-CoA-transferase (*SL1344_3092*, *S*.En *cat2*, *Yersinia y2395/ripA*; Figure S5; [60]). Biochemical data and structural information suggest that this genomic island may generate a CoA-derivative and an acyl carboxylic acid, thus contributing to bacterial metabolism during infection [61]–[63]. Indeed, *S*. Typhimurium is known to express *SL1344_3093* when residing within macrophages [63], [64].

Furthermore, mutations in *SL1344_3093* and/or genes of the conserved locus were found to attenuate wt *S*. Typhimurium infection in a mouse model for typhoid fever [65], *S*. Gallinarum infection in a chicken model and the growth of *S*. Enteritidis, *S*. Gallinarum and *Y. pestis* within macrophages [60], [66], [67]. Based on these findings, we speculate that the LAV strain *ssaV* may require *SL1344_3093* for metabolism in order to colonize a certain (unidentified) phagocyte population in *cybb* ^−/−^ *nos2* ^−/−^ mice, e.g. by providing some essential metabolite. In wt C57BL/6 mice, this phenotype would not be observed, because *cybb*/*nos2*-mediated defenses would kill the LAV (*ssaV* and Z234, alike) in this cell type.

Other authors have suggested that *SL1344_3093* shares similarity with monoamine oxidases, enzymes converting amino acetone (e.g. derived from L-threonine) into methyl glyoxal (MG) [68]. Methyl glyoxal, although toxic, has been proposed as a necessary metabolite under conditions of phosphate starvation or carbon super sufficiency (accumulation of di-hydroxy acetone phosphate). As intracellular *Salmonella* are known to be starved for phosphate, this may suggest a metabolic requirement for *SL1344_3093* in intracellular growth and/or survival [64], [69]. Possibly, methylglyoxal can be detoxified via the putative lactoyl glutathione lyase *SL1344_3091*, which may convert MG into S-lactoyl glutathione. Interestingly, unlike the other co-regulated genes (3094-3092), *SL1344_3091* is absent from *Mycobacteria* spp or *Yersinia* spp. Neverthless, is found in *S*. Enteritidis suggesting that Non-typhoidal *Salmonella* spp. might have evolved further to adapt to an intracellular life cycle inside phagocytic cells. A second possibility may arise from work in *Klebsiella aerogenes*, where an open reading frame similar to *SL1344_3093* was found to oxidize several monoamines to their corresponding aldehydes, for example, tyramine (derivative of tyrosine) to *p*-hydroxyphenyl acetaldehyde [70], [71]. Thus, the exact mechanism explaining why *SL1344_3094*-*SL1344_3091* enhances within-macrophage growth remains unclear. *Y. pestis ripA* and *ripB* (the *SL1344_3093* homolog) mutants grow poorly in wild type, but not in *nos2* ^−/−^ macrophages [60]. It was speculated that the *ripABC* operon may lower production of reactive nitrogen species, a potent antimicrobial defense of macrophages (Pujol et al., 2005; Torres et al., 2011). Clearly, this cannot explain the super-attenuation phenotype of *S*. Tm *ssaV SL1344_3093* observed in the *cybb* ^−/−^ *nos2* ^−/−^ mice. Identifying the molecular basis of LAV super-attenuation in immuno-deficient hosts will be an interesting topic for future research.

In conclusion, incorporating additional “safety” features such as the *SL1344_3093* mutation identified in our study may be of general interest for the design of new LAV generations. We propose that the systematic screening for attenuated mutants in immune compromised experimental host systems may be an efficient way to identify suitable target genes which could then be disrupted in experimental, or even in the existing LAV. This may help to reduce the risk of fulminant vaccine strain infections, i.e. in immuno-compromised hosts present in the general target population.

## Materials and Methods

### Bacteria and plasmids

*ssaV* (M2730, SL1344 *ΔssaV*) was created by phage transduction of *ssaV::aphT* [65] into *S.* Typhimurium SL1344 (SB300) [72] and flip-mediated removal of the *aphT* cassette. 35 additional *geneX::aphT* alleles from a systematic mutant collection [65] were transduced into M2730 and the kanamycin cassette was removed via flip recombinase. Finally, one out of 7 different WITS tags (unique sequence + kanamycin resistance cassette; inserted in a pseudogene region [73]), was transduced yielding the strains shown in Table S1. M2735 (SL1344; *ssaV::cat*, WITS-17) was created equivalently using the *ssaV::cat* allele from M1516 (*ssaV::cat*) [74]. *S*. Enteritidis M1525 and *S*. Typhimurium SKI12 (SL1344, Δ*wbaP*) have been described [75], [76]. For infections, bacteria were grown over night, diluted by 1∶20 in fresh LB (0.3 M NaCl) and sub cultured for another 4 h at 37°C under mild aeration [31].

*SL1344_3093* was amplified by PCR using the following primers (GATTCATATGGATGATAGATGAAGCCGTTGCCCGTCAGGCCCGGAGATTACTCG; CATTATAGCGGCCGCGCATTCAGCAAAGCTGGAGGCTCTGCTGCAAACATTCCCATAG), subcloned into pCR®2.1-TOPO® (Invitrogen™), sequenced and moved via *Xba*I/*Sac*I into pWKS30 [77], under control of the *lac* promoter, yielding pZ200 (p_compl._).

### Mice

All mice were specific pathogen free (SPF) and were maintained in individually ventilated cages (IVC). Wild type, *cybb^−/−^/nos2* ^−/−^ (B6.129S6-*Cybb^tm1Din^*/J40 and B6;129P2-*Nos2^tm1Lau^*/J41, [78]), *tnfr1* ^−/−^ (B6-TNFR1^tm^), caspase1^−/−^ ((B6;129S2-Casp1^tm1Sesh^) [79]) and *rag1* ^−/−^ [38] mice were bred in the C57BL/6 background (highly susceptible to systemic *S*. Typhimurium infection due to the *Nramp1* (*slc11a1*) mutation [80] at the Rodent Center HCI (RCHCI, Zürich). *Ifng1* ^−/−^, *c*3^−/−^ knockouts (generous gift from M. Kopf; BioSupport AG, Schlieren) were in the BALB/c background.

### Ethics statement

All animal experiments were approved by the authorities (Kantonales Veterinäramt Zürich, license: 223/2010) and performed according to local guidelines (TschV, Zurich) and the Swiss animal protection law (TschG).

### Streptomycin mouse model

Mice were pretreated with streptomycin (20 mg, by gavage) 24 h prior to infection (5×10^7^ cfu by gavage, if not stated otherwise) as described [31]. Fecal shedding and bacterial loads in the cecum content and the indicated organs were determined at the indicated time points p.i. by plating on MacConkey agar supplemented with the appropriate antibiotics. For statistical analysis, samples w/o bacteria were adjusted to the minimal detectable level (10 CFU/organ in the MLN, 20 CFU/organ in the spleen, 10/× cfu/g of cecum content or feces; x = weight of the fecal sample).

Cecal inflammation was scored as described previously [81]. Briefly, 5 µm sections of cryo-embedded tissue were stained with H&E and scored for sub-mucosal edema (0–3), PMN infiltration (0–4), loss of goblet cells (0–3) and epithelial ulceration (0–3) with a total score that range between 0–13.

### Vaccination/challenge protocol

The vaccination/challenge protocol has been described [28], [29]. Streptomycin treated mice were immunized with Z234, M2735 (5×10^7^ cfu by gavage) or PBS. Fecal samples were collected, weekly. At day 40 p.i., we analyzed mucosal histopathology and organ loads (see above). Remaining animals were treated with ampicillin (20 mg by gavage) and infected 24 h later with wt *S*. Typhimurium SL1344 (200 cfu by gavage). At day 2 p.i., we assessed colonization by the challenge strain and mucosal inflammation. Antibody samples were analyzed from serum or gut washes obtained post mortem (sIgA).

Antibody responses were quantified by bacterial FACS and by Western blot, as described [28], [29]. Briefly, for bacterial FACS, Z234 cells were washed (PBS, 2% BSA, 2% NaN_3_) and incubated (30 min, 4°C) with different dilutions of the respective serum (heat inactivated; 1∶20, 1∶60, 1∶120) or gut wash sample (undiluted; 1∶3, 1∶9). After washing, the coating mouse antibodies were detected using PE-anti-mouse IgG (dilution, 1∶100; Jackson Immunoresearch Europe), APC-anti mouse-IgM (dilution, 1∶100; BD Pharmingen) or FITC-anti mouse IgA (dilution, 1∶50; BD Pharmingen; 30 min, 4°C). After fixation (PBS, 2% paraformaldehyde), the bacterial surface staining was quantified by FACS (FlowJo software 7.6.3, Treestar, USA [29]).

### Statistical analysis

Statistical analyses were performed using the exact Mann-Whitney U test (SPSS, version 14.0.). P values<.05 (two tailed) were considered statistically significant.

## Supporting Information

Figure S1Screening protocol. Mutant strains were screened in streptomycin pretreated *cybb* ^−/−^ *nos2* ^−/−^ mice (see, above; 3 mice per group of mutants). The inoculum was composed of six double mutants and strain M2735 ( = wt control) yielding a 1∶1∶1∶1∶1∶1∶1 mixture (5×10^7^ cfu total in 50 µl PBS). Importantly, each of the strains harbored a kanamycin resistance cassette and a unique WITS-sequence. At day 4 p.i., cecal contents, MLN, spleen and liver were sampled and re-suspended in 500 µl or 1 ml of PBS (0.5% BSA, 0.5% tergitol) and homogenized using a bead beater (Qiagen). 50% of the tissue homogenates were used for an enrichment culture in LB broth (5 ml), recovery of the bacteria by centrifugation and extraction of bacterial DNA via the Qiagen DNA mini kit. The relative abundance of the different strains was determined via real time PCR quantification of the WITS tag sequences, as described [37]. The net bacterial load (total cfu of all bacterial mutants taken together; C) for a given organ was determined by plating the remaining 50% of the homogenate on MacConkey agar (50 µg/µl kanamycin). Relative cfu (R_cfu_) of every given mutant was calculated as: 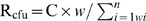; w: amplified RT PCR signal of any given mutant carrying a unique WITS tag. C: net bacterial load/organ. A typical data set is shown in Figure 1.(TIF)

Figure S2 Competitive infections with Z234 and *ssaV*. (A) *cybb* ^−/−^ *nos2* ^−/−^ mice (n = 6) or (B) C57BL/6 mice (n = 5) were infected with a 1∶1 mixture of both strains (5×10^7^ cfu in total by gavage) and analyzed as described above (Figure 1, 2). The competitive index was defined as the relative ratio of the real time PCR-amplified DNA signal of the 2 mutants (Z234 and M2735) to that of the initial ratio of the same 2 mutants in the inoculum. Median of the competitive indices of the corresponding mice group was plotted with error bars indicating standard deviation; *, statistically significant (p<.05; Mann-Whitney U-test).
(TIF)

Figure S3 Fecal shedding and disease parameter data as control for the vaccination experiment shown in Figure 3. i.e., Vaccination-challenge experiments that display the immunogenic potential of Z234. For vaccination, C57BL/6 mice were inoculated with PBS (n = 4; empty symbols), *ssaV* (5×10^7^ cfu; n = 9; grey symbols) or Z234 (5×10^7^ cfu; n = 13; black symbols). (A) Fecal shedding as analyzed by plating. PBS-controls: below detection limit (striped line); (B) Colonization by the vaccine strain and cecal pathology (scale 0–13; score ≤3 considered not inflamed [38], [53]) at day 40 post vaccination (M2735, n = 4; Z234, n = 5 mice). N.s.: not significant; *, statistically significant (p<.05; Mann-Whitney U-test).
(TIF)

Figure S4 Cecal pathology of mock vaccinated (PBS) versus Z234 vaccinated mice, day 2 post challenge. (A) Hematoxylin and Eosin (H & E) staining of representative mock vaccinated and wt challenged mouse, day 2 post challenge was shown (1×, 10× and 20×). (B) H & E staining of representative Z234 vaccinated and wt challenged mice, day 2 post challenge was shown.
(TIF)

Figure S5 Features of the *SL1344_3093* locus. (A) tRNA^pheV^ locus of *S*. Typhimurium SL1344. The graph depicts the gene neighborhood architechture of *SL1344_3093* as per *S*. Typhimurium genome sequence, FQ312003.1. Both experimentally observed and predicted functions of the genes in the 3093 neighborhood are outlined. (B) SL1344 tRNA^pheV^ homology locus. The graph depicts the *SL1344 3093* gene neighborhood homology in other related pathogens; *Mycobacterium tuberculosis* H37rV, *Y. pestis* KIM10 and to other *S. enterica* sub species I serovars; *S*. Enteritidis and *S*. Gallinarum. (C). Predicted functional partners of *SL1344_3093* (*STM3119*) as determined by STRING database (confidence view), version 9.0. (STRING database annotation was based on *S*. Typhimurium LT2 genome). The relative thickness of the edges (signify a functional association based on one or more parameters; Neighborhood, Gene Fusion, Cooccurrence, Coexpression, Experimental findings, Databases, Text mining, Homology) between the nodes (genes) represent degree of confidence in functional partner prediction.
(TIF)

Table S1 List of 35 *ssaV* double mutants, rank ordered according to their degree of attenuation in spleens of *cybb* ^−/−^ *nos2* ^−/−^ mice, day 4 p.i. Pools of *ssaV* double mutants (5–6 mutants and a spike-in control, i.e. the parent strain *ssaV*) were used to coinfect *cybb* ^−/−^ *nos2* ^−/−^ mice (3 independent mice). At day 4 p.i., organs were extracted, enriched for bacterial DNA and the relative abundance of a given bacterial mutant was analyzed by real-time PCR using WITS-specific primers. The real-time PCR signal of a given bacterial mutant was normalized relative to the mean amplified signal of all mutants and their abundance relative to the inoculum was presented as the attenuation score.
(PDF)

Reference S1 Clinical cases of invasive non-typhoidal salmonellosis. Case reports of bacteremia due to invasive disease caused by non-typhoidal *Salmonella* spp., *S*. Typhimurium and *S*. Enteritidis, with a major focus in children and HIV infected people. The list is non-comprehensive.
(DOCX)
