# Supplementary figures and images for: Live Attenuated S. Typhimurium Vaccine with Improved Safety in Immuno-Compromised Mice
Source: PLoS One. 2012 Sep 24;7(9):e45433. doi: 10.1371/journal.pone.0045433 (PMC3454430; doi:10.1371/journal.pone.0045433)

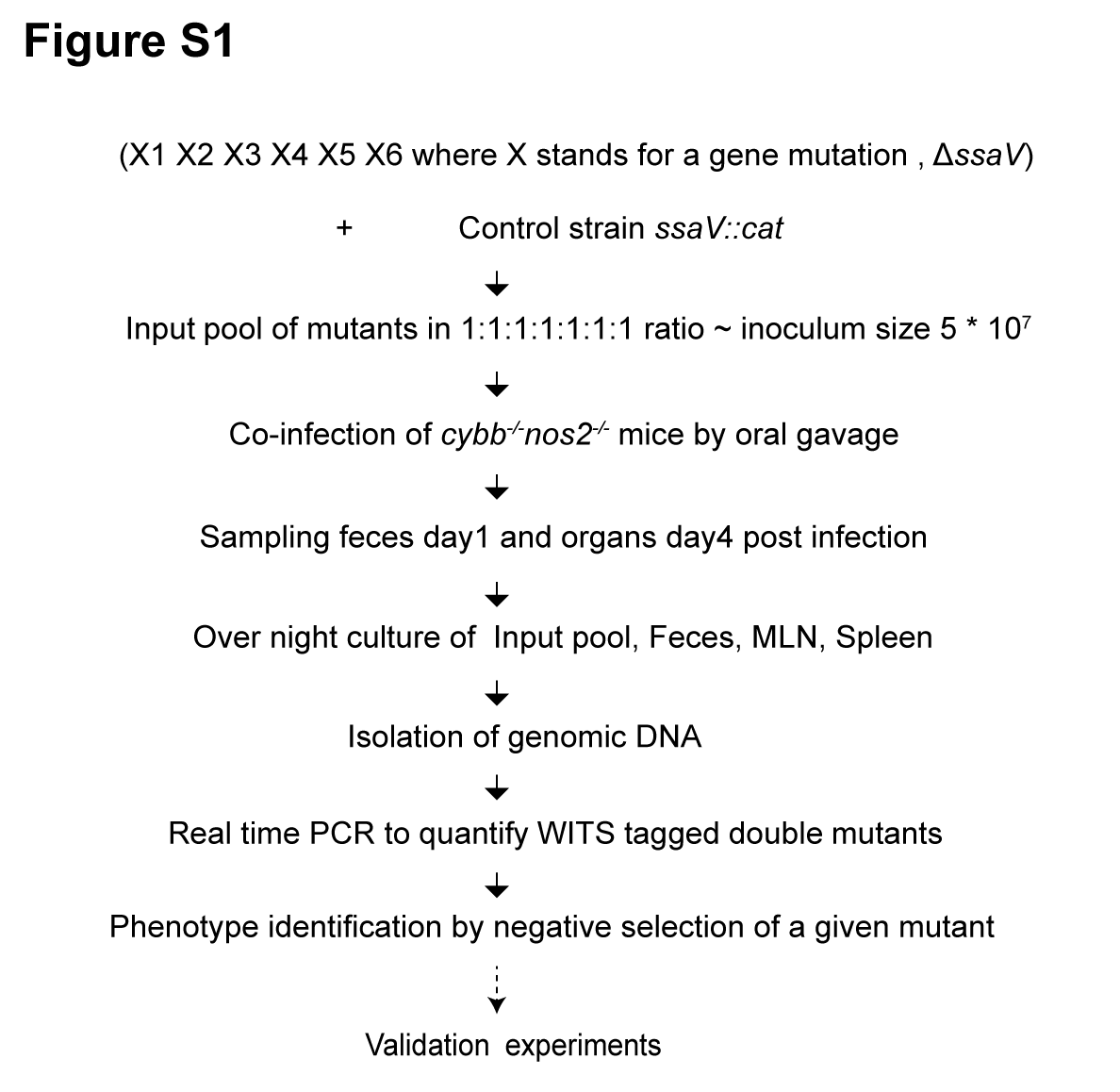

Supplement: Figure S1 — Screening protocol. Mutant strains were screened in streptomycin pretreated cybb −/− nos2 −/− mice (see, above; 3 mice per group of mutants). The inoculum was composed of six double mutants and strain M2735 ( = wt control) yielding a 1∶1∶1∶1∶1∶1∶1 mixture (5×10^7^ cfu total in 50 µl PBS). Importantly, each of the strains harbored a kanamycin resistance cassette and a unique WITS-sequence. At day 4 p.i., cecal contents, MLN, spleen and liver were sampled and re-suspended in 500 µl or 1 ml of PBS (0.5% BSA, 0.5% tergitol) and homogenized using a bead beater (Qiagen). 50% of the tissue homogenates were used for an enrichment culture in LB broth (5 ml), recovery of the bacteria by centrifugation and extraction of bacterial DNA via the Qiagen DNA mini kit. The relative abundance of the different strains was determined via real time PCR quantification of the WITS tag sequences, as described [37]. The net bacterial load (total cfu of all bacterial mutants taken together; C) for a given organ was determined by plating the remaining 50% of the homogenate on MacConkey agar (50 µg/µl kanamycin). Relative cfu (Rcfu) of every given mutant was calculated as: ; w: amplified RT PCR signal of any given mutant carrying a unique WITS tag. C: net bacterial load/organ. A typical data set is shown in Figure 1. (TIF) [file pone.0045433.s001.tif]

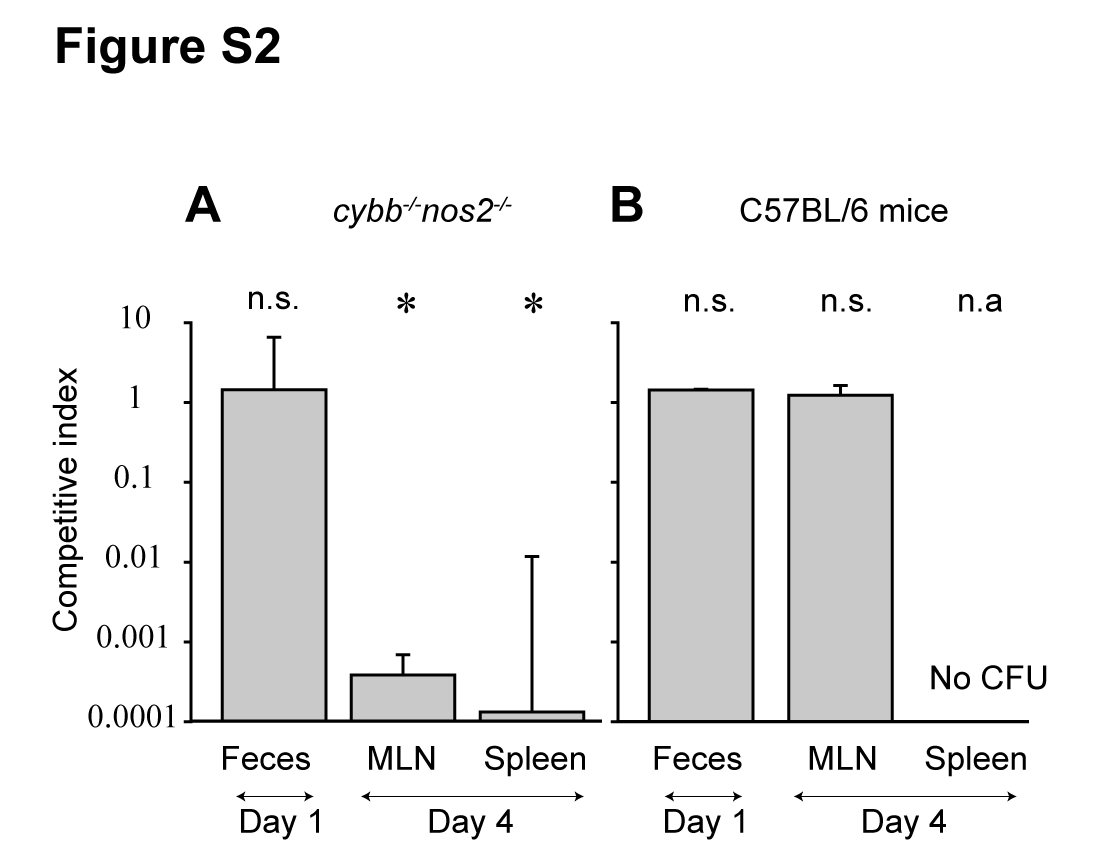

Supplement: Figure S2 — Competitive infections with Z234 and ssaV. (A) cybb −/− nos2 −/− mice (n = 6) or (B) C57BL/6 mice (n = 5) were infected with a 1∶1 mixture of both strains (5×10^7^ cfu in total by gavage) and analyzed as described above (Figure 1, 2). The competitive index was defined as the relative ratio of the real time PCR-amplified DNA signal of the 2 mutants (Z234 and M2735) to that of the initial ratio of the same 2 mutants in the inoculum. Median of the competitive indices of the corresponding mice group was plotted with error bars indicating standard deviation; *, statistically significant (p<.05; Mann-Whitney U-test). (TIF) [file pone.0045433.s002.tif]

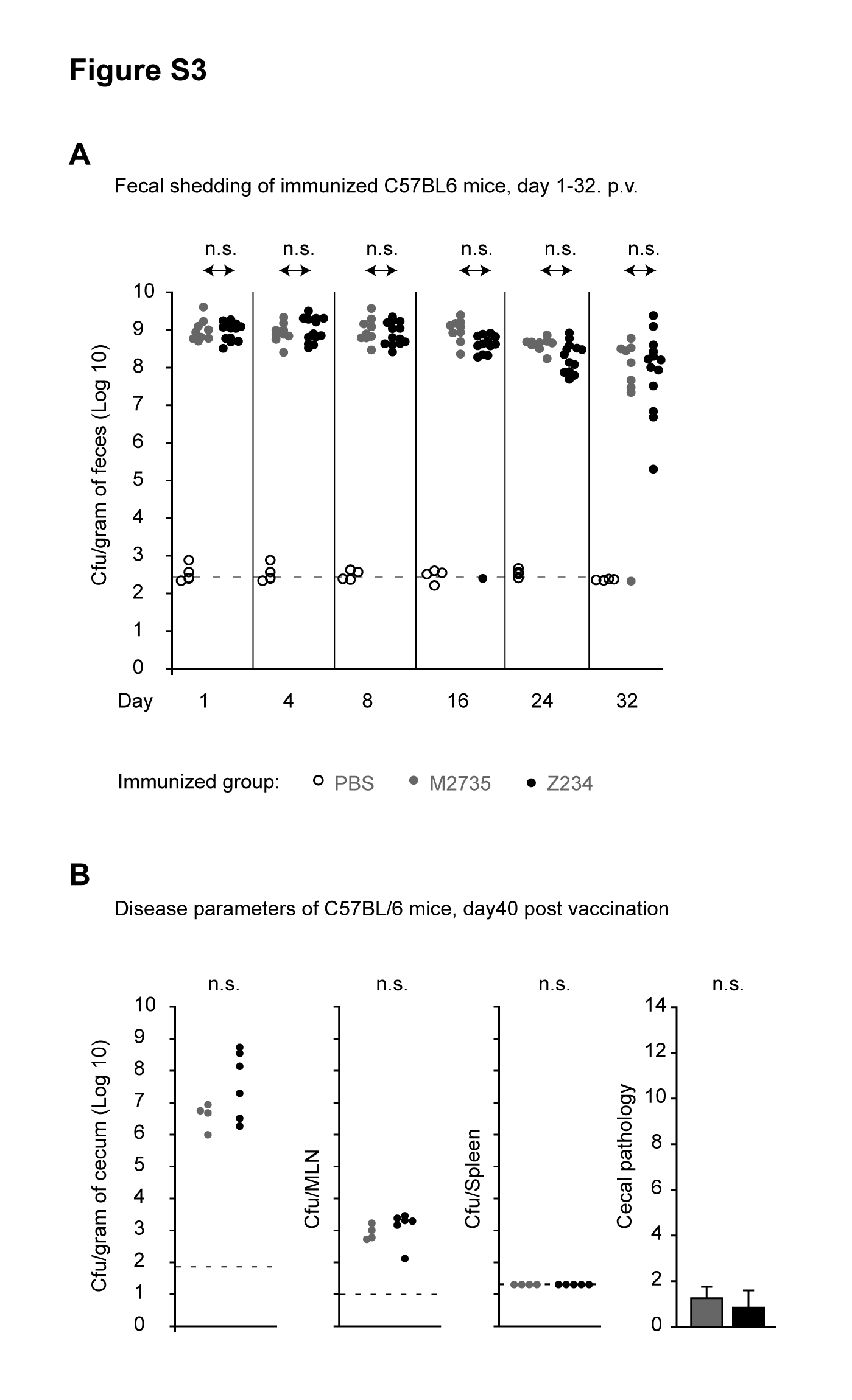

Supplement: Figure S3 — Fecal shedding and disease parameter data as control for the vaccination experiment shown in Figure 3. i.e., Vaccination-challenge experiments that display the immunogenic potential of Z234. For vaccination, C57BL/6 mice were inoculated with PBS (n = 4; empty symbols), ssaV (5×10^7^ cfu; n = 9; grey symbols) or Z234 (5×10^7^ cfu; n = 13; black symbols). (A) Fecal shedding as analyzed by plating. PBS-controls: below detection limit (striped line); (B) Colonization by the vaccine strain and cecal pathology (scale 0–13; score ≤3 considered not inflamed [38], [53]) at day 40 post vaccination (M2735, n = 4; Z234, n = 5 mice). N.s.: not significant; *, statistically significant (p<.05; Mann-Whitney U-test). (TIF) [file pone.0045433.s003.tif]

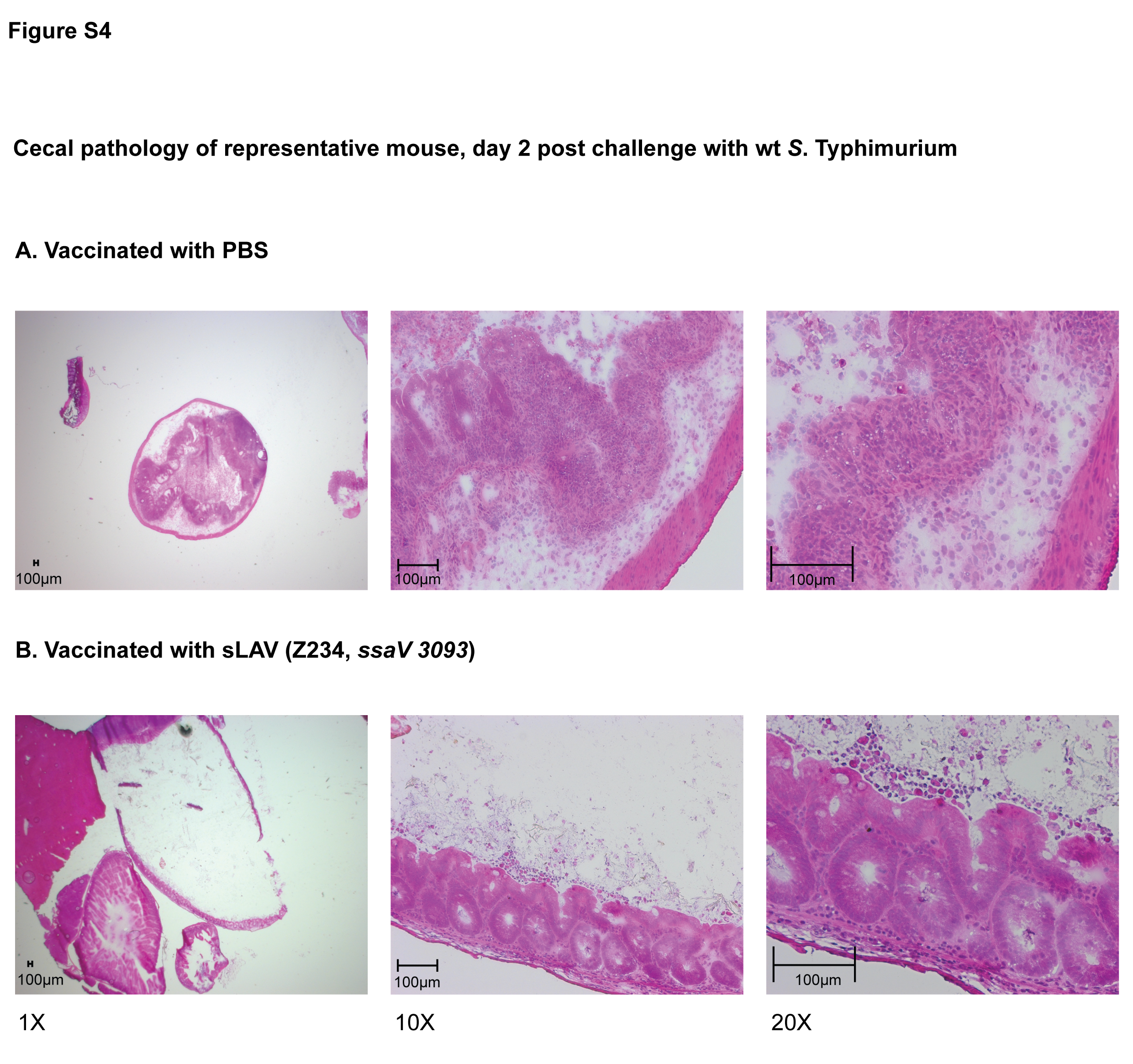

Supplement: Figure S4 — Cecal pathology of mock vaccinated (PBS) versus Z234 vaccinated mice, day 2 post challenge. (A) Hematoxylin and Eosin (H & E) staining of representative mock vaccinated and wt challenged mouse, day 2 post challenge was shown (1×, 10× and 20×). (B) H & E staining of representative Z234 vaccinated and wt challenged mice, day 2 post challenge was shown. (TIF) [file pone.0045433.s004.tif]

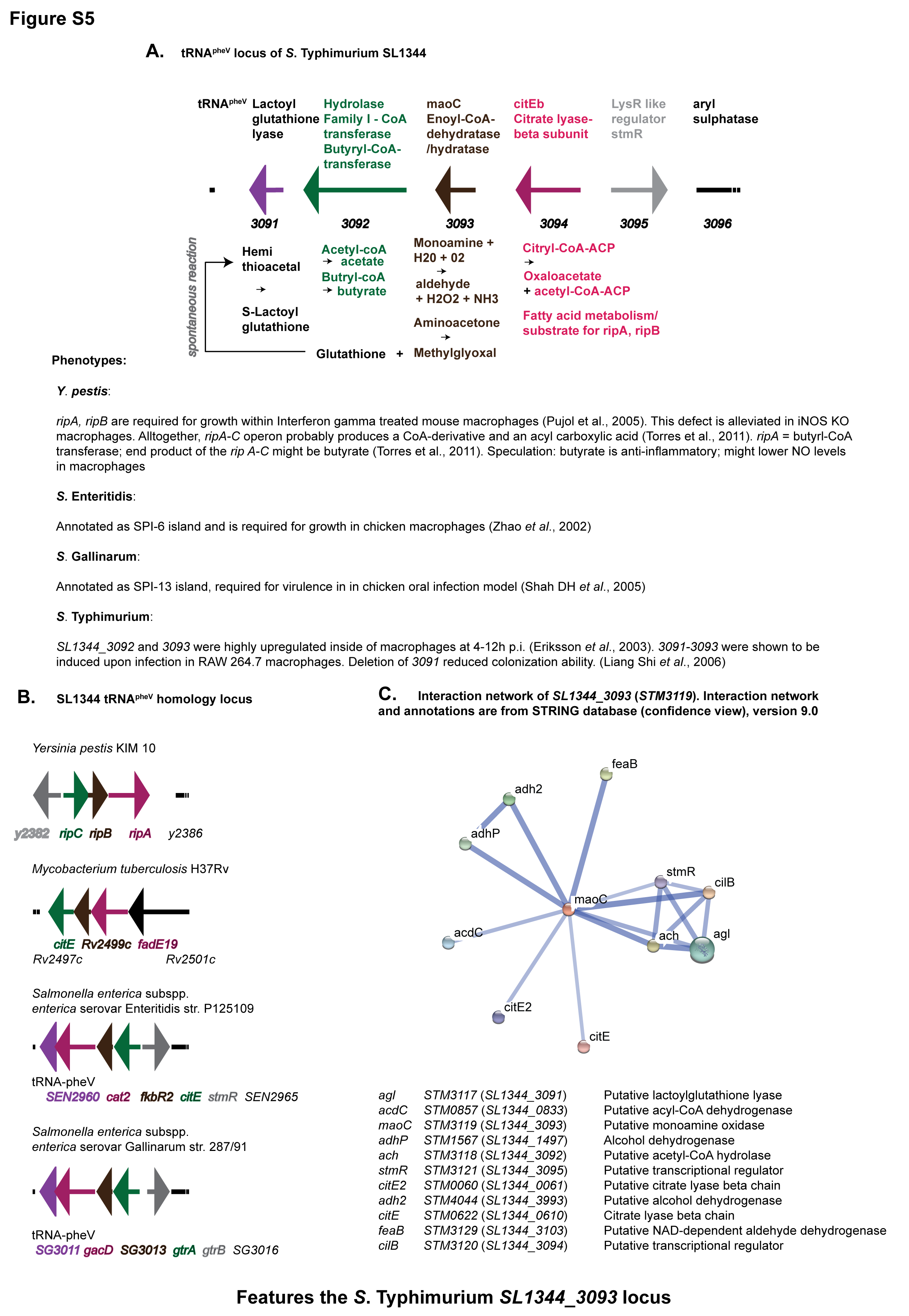

Supplement: Figure S5 — Features of the SL1344_3093 locus. (A) tRNApheV locus of S. Typhimurium SL1344. The graph depicts the gene neighborhood architechture of SL1344_3093 as per S. Typhimurium genome sequence, FQ312003.1. Both experimentally observed and predicted functions of the genes in the 3093 neighborhood are outlined. (B) SL1344 tRNApheV homology locus. The graph depicts the SL1344 3093 gene neighborhood homology in other related pathogens; Mycobacterium tuberculosis H37rV, Y. pestis KIM10 and to other S. enterica sub species I serovars; S. Enteritidis and S. Gallinarum. (C). Predicted functional partners of SL1344_3093 (STM3119) as determined by STRING database (confidence view), version 9.0. (STRING database annotation was based on S. Typhimurium LT2 genome). The relative thickness of the edges (signify a functional association based on one or more parameters; Neighborhood, Gene Fusion, Cooccurrence, Coexpression, Experimental findings, Databases, Text mining, Homology) between the nodes (genes) represent degree of confidence in functional partner prediction. (TIF) [file pone.0045433.s005.tif]
