# Supplementary material for: Live Attenuated S. Typhimurium Vaccine with Improved Safety in Immuno-Compromised Mice
Source: PLoS One. 2012 Sep 24;7(9):e45433. doi: 10.1371/journal.pone.0045433 (PMC3454430; doi:10.1371/journal.pone.0045433)
Supplement: Table S1 — List of 35 ssaV double mutants, rank ordered according to their degree of attenuation in spleens of cybb −/− nos2 −/− mice, day 4 p.i. Pools of ssaV double mutants (5–6 mutants and a spike-in control, i.e. the parent strain ssaV) were used to coinfect cybb −/− nos2 −/− mice (3 independent mice). At day 4 p.i., organs were extracted, enriched for bacterial DNA and the relative abundance of a given bacterial mutant was analyzed by real-time PCR using WITS-specific primers. The real-time PCR signal of a given bacterial mutant was normalized relative to the mean amplified signal of all mutants and their abundance relative to the inoculum was presented as the attenuation score. (PDF) [file pone.0045433.s006.pdf]

Table. S1. The set of *S.* Typhimurium *ssaV* double mutants which were screened for attenuation in cybb/nos2$^{-/-}$ mice.

#	SL1344 Identifier	LT2 ortholog	WITS tag	Gene Name	Description as per SL1344 genome reference NC_016810.1	Abundance of mutants relative to mean inoculum	Specific attenuation of mutants in mice relative to inoculum		
							Day1 feces	MLN	Spleen
1*	SL1344_3093	**STM3119**	**WITS21**	**SL1344_3093**	**Hypothetical protein**	**1.13**	**1.09**	**0.001**	**0.001**
2	SL1344_0466	STM0473	WITS19	*hha*	Hemolysin expression modulating protein	0.36	0.003	0.14	0.004
3	SL1344_1031	STM1092	WITS13	SL1344_1031	Hypothetical protein	0.69	0.10	0.10	0.03
4	SL1344_1180	STM1241	WITS1	*msgA*	Putative virulence protein MsgA	0.76	0.08	0.06	0.03
5	SL1344_1181	STM1242	WITS21	*envE*	Putative lipoprotein	0.68	0.10	0.10	0.05
6	SL1344_1026	STM1087	WITS2	*pipA*	Hypothetical protein	0.64	0.10	0.07	0.05
7	SL1344_2579	STM2617	WITS13	SL1344_2579	Putative antitermination protein	0.91	0.06	0.10	0.05
8	SL1344_0675	STM0693	WITS1	*fur*	Ferric uptake regulation protein	0.54	0.03	0.22	0.06
9	SL1344_2525	STM2563	WITS19	*yfhG*	Hypothetical protein	0.66	0.07	0.08	0.09
10	SL1344_2363	STM2395	WITS1	*pgtE*	Outer membrane protease E	0.99	0.14	0.22	0.24
11	SL1344_2209	STM2233	WITS19	SL1344_2209	Tail fiber assembly protein	0.69	0.66	0.55	0.25
12	SL1344_1033	STM1094	WITS11	*pipD*	Putative secreted peptidase	2.85	0.22	0.23	0.28
13	SL1344_2841	STM2861	WITS13	*sitA*	Iron transport protein, periplasmic-binding protein	1.11	1.41	0.48	0.37
14	SL1344_1372	STM1440	WITS11	*sodC*	Copper/zinc superoxide dismutase	1.38	1.16	0.59	0.54
15	SL1344_1467	STM1538	WITS13	*hyaB2*	Uptake hydrogenase-1 large subunit	0.89	1.20	1.06	0.60
16	SL1344_2273	STM2304	WITS21	*pmrD*	Polymyxin B resistance protein	1.40	1.54	0.90	0.65
17	SL1344_2976	STM2998	WITS2	SL1344_2976	Hypothetical protein	0.92	1.16	1.10	0.71
18	SL1344_2532	STM2570	WITS19	STM2570	Putative PTS system IIBC component	0.98	0.94	0.85	0.74
19	SL1344_2802	STM2817	WITS21	*luxS*	S-ribosylhomocysteinase	1.11	1.48	1.02	0.75
20	SL1344_1983	STM2008	WITS11	SL1344_1983	Hypothetical protein	1.36	0.95	0.88	0.80
21	SL1344_2208	STM2232	WITS13	SL1344_2208	Putative lipopolysaccharide modification acyltransferase	1.05	0.94	1.07	0.87
22	SL1344_1982	STM2007	WITS2	SL1344_1982	Hypothetical protein	1.09	0.97	1.02	0.87
23	SL1344_2966	STM2986	WITS19	SL1344_2966	Hypothetical protein	1.07	1.04	0.99	0.92
24	SL1344_1979	STM2005	WITS1	SL1344_1979	Hypothetical protein	1.23	0.96	0.87	0.94
25	SL1344_1987	STM2011	WITS11	SL1344_1987	Hypothetical protein	1.31	0.97	1.15	1.01
26	SL1344_1195	STM1259	WITS2	SL1344_1195	Putative ABC transport ATP-binding subunit	0.85	1.43	1.54	1.06
27	SL1344_4253	STM4317	WITS11	SL1344_4253	Hypothetical protein	1.24	1.43	1.12	1.06
28	SL1344_2386	STM2423	WITS1	*yfeN*	Hypothetical protein	1.19	1.39	1.39	1.22
29	SL1344_1879	STM1950	WITS19	*sdiA*	Transcriptional regulator of ftsQAZ gene cluster (LuxR/UhpA family)	1.01	1.02	1.23	1.26
30	SL1344_2207	STM2231	WITS13	SL1344_2207	Putative SsrB-regulated factor	0.75	0.85	1.63	1.32
31	SL1344_3311	STM3339	WITS11	*nanA*	N-acetylneuraminate lyase	1.03	0.68	0.82	1.32
32	SL1344_1688	STM1760	WITS21	SL1344_1688	Hypothetical protein	1.20	1.15	1.19	1.58
33	SL1344_2578	STM2616	WITS1	SL1344_2578	Bacteriophage protein	1.03	1.07	1.17	1.75
34	SL1344_1198	STM1263	WITS2	SL1344_1198	Hypothetical protein	1.05	1.14	1.26	1.90
35	SL1344_1376	STM1444	WITS2	*slyA*	Transcriptional regulator slyA	0.87	0.24	3.59	4.69

# Strains were ordered according to their degree of attenuation in spleen, day 4 post infection.
* This mutation is present in the sLAV strain Z234
The isogenic control strain *ssaV::cat* (WITS tag 17; M2735) was always included as internal reference
