# Supplementary material for: Live Attenuated S. Typhimurium Vaccine with Improved Safety in Immuno-Compromised Mice
Source: PLoS One. 2012 Sep 24;7(9):e45433. doi: 10.1371/journal.pone.0045433 (PMC3454430; doi:10.1371/journal.pone.0045433)
Supplement: Reference S1 — Clinical cases of invasive non-typhoidal salmonellosis. Case reports of bacteremia due to invasive disease caused by non-typhoidal Salmonella spp., S. Typhimurium and S. Enteritidis, with a major focus in children and HIV infected people. The list is non-comprehensive. (DOCX) [file pone.0045433.s007.docx]

**Clinical cases of invasive non-typhoidal salmonellosis**

1. Abramson S, Kramer SB, Radin A, Holzman R (1985) Salmonella bacteremia in systemic lupus erythematosus. Eight-year experience at a municipal hospital. Arthritis Rheum 28: 75-79.

2. Berkley JA, Lowe BS, Mwangi I, Williams T, Bauni E, et al. (2005) Bacteremia among children admitted to a rural hospital in Kenya. N Engl J Med 352: 39-47.

3. Brent AJ, Oundo JO, Mwangi I, Ochola L, Lowe B, et al. (2006) Salmonella bacteremia in Kenyan children. Pediatr Infect Dis J 25: 230-236.

4. Cherubin CE, Neu HC, Imperato PJ, Harvey RP, Bellen N (1974) Septicemia with non-typhoid salmonella. Medicine (Baltimore) 53: 365-376.

5. Chierakul W, Rajanuwong A, Wuthiekanun V, Teerawattanasook N, Gasiprong M, et al. (2004) The changing pattern of bloodstream infections associated with the rise in HIV prevalence in northeastern Thailand. Trans R Soc Trop Med Hyg 98: 678-686.

6. Cohen JI, Bartlett JA, Corey GR (1987) Extra-intestinal manifestations of salmonella infections. Medicine (Baltimore) 66: 349-388.

7. Enwere G, Biney E, Cheung YB, Zaman SM, Okoko B, et al. (2006) Epidemiologic and clinical characteristics of community-acquired invasive bacterial infections in children aged 2-29 months in The Gambia. Pediatr Infect Dis J 25: 700-705.

8. Galofre J, Moreno A, Mensa J, Miro JM, Gatell JM, et al. (1994) Analysis of factors influencing the outcome and development of septic metastasis or relapse in Salmonella bacteremia. Clin Infect Dis 18: 873-878.

9. Gilks CF, Brindle RJ, Otieno LS, Simani PM, Newnham RS, et al. (1990) Life-threatening bacteraemia in HIV-1 seropositive adults admitted to hospital in Nairobi, Kenya. Lancet 336: 545-549.

10. Gordon MA, Banda HT, Gondwe M, Gordon SB, Boeree MJ, et al. (2002) Non-typhoidal salmonella bacteraemia among HIV-infected Malawian adults: high mortality and frequent recrudescence. AIDS 16: 1633-1641.

11. Gordon MA, Kankwatira AM, Mwafulirwa G, Walsh AL, Hopkins MJ, et al. (2010) Invasive non-typhoid salmonellae establish systemic intracellular infection in HIV-infected adults: an emerging disease pathogenesis. Clin Infect Dis 50: 953-962.

12. Graham SM, Walsh AL, Molyneux EM, Phiri AJ, Molyneux ME (2000) Clinical presentation of non-typhoidal Salmonella bacteraemia in Malawian children. Trans R Soc Trop Med Hyg 94: 310-314.

13. Han T, Sokal JE, Neter E (1967) Salmonellosis in disseminated malignant diseases. A seven-- year review (1959-1965). N Engl J Med 276: 1045-1052.

14. Hsu RB, Lin FY (2008) Nontyphoid Salmonella infection in heart transplant recipients. Am J Med Sci 336: 393-396.

15. Ikumapayi UN, Antonio M, Sonne-Hansen J, Biney E, Enwere G, et al. (2007) Molecular epidemiology of community-acquired invasive non-typhoidal Salmonella among children aged 2 29 months in rural Gambia and discovery of a new serovar, Salmonella enterica Dingiri. J Med Microbiol 56: 1479-1484.

16. Kariuki S, Revathi G, Kariuki N, Kiiru J, Mwituria J, et al. (2006) Characterisation of community acquired non-typhoidal Salmonella from bacteraemia and diarrhoeal infections in children admitted to hospital in Nairobi, Kenya. BMC Microbiol 6: 101.

17. Lepage P, Bogaerts J, Van Goethem C, Ntahorutaba M, Nsengumuremyi F, et al. (1987) Community-acquired bacteraemia in African children. Lancet 1: 1458-1461.

18. Mandomando I, Macete E, Sigauque B, Morais L, Quinto L, et al. (2009) Invasive non-typhoidal Salmonella in Mozambican children. Trop Med Int Health 14: 1467-1474.

19. Obaro S, Lawson L, Essen U, Ibrahim K, Brooks K, et al. (2011) Community acquired bacteremia in young children from central Nigeria--a pilot study. BMC Infect Dis 11: 137.

20. O'Dempsey TJ, McArdle TF, Lloyd-Evans N, Baldeh I, Laurence BE, et al. (1994) Importance of enteric bacteria as a cause of pneumonia, meningitis and septicemia among children in a rural community in The Gambia, West Africa. Pediatr Infect Dis J 13: 122-128.

21. Pablos JL, Aragon A, Gomez-Reino JJ (1994) Salmonellosis and systemic lupus erythematosus. Report of ten cases. Br J Rheumatol 33: 129-132.

22. Peters RP, Zijlstra EE, Schijffelen MJ, Walsh AL, Joaki G, et al. (2004) A prospective study of bloodstream infections as cause of fever in Malawi: clinical predictors and implications for management. Trop Med Int Health 9: 928-934.

23. Ramos JM, Garcia-Corbeira P, Aguado JM, Plaza JJ, Soriano F (1995) Nontyphoid Salmonella extraintestinal infections in renal transplant recipients. Nephron 71: 489-490.

24. Sigauque B, Roca A, Mandomando I, Morais L, Quinto L, et al. (2009) Community-acquired bacteremia among children admitted to a rural hospital in Mozambique. Pediatr Infect Dis J 28: 108-113.

25. Walsh AL, Phiri AJ, Graham SM, Molyneux EM, Molyneux ME (2000) Bacteremia in febrile Malawian children: clinical and microbiologic features. Pediatr Infect Dis J 19: 312-318.
